# Short halt in vaping modifies cardiorespiratory parameters and urine metabolome: a randomized trial

**DOI:** 10.1152/ajplung.00268.2019

**Published:** 2019-11-13

**Authors:** Martin Chaumont, Vanessa Tagliatti, El Mehdi Channan, Jean-Marie Colet, Alfred Bernard, Sofia Morra, Guillaume Deprez, Alain Van Muylem, Nadia Debbas, Thomas Schaefer, Vitalie Faoro, Philippe van de Borne

**Affiliations:** ^1^Department of Cardiology, Erasme University Hospital, Université Libre de Bruxelles, Brussels, Belgium; ^2^Institute for Translational Research in Cardiovascular and Respiratory Sciences, Université Libre de Bruxelles, Brussels, Belgium; ^3^Department of Human Biology and Toxicology, University of Mons, Mons, Belgium; ^4^Laboratory of Toxicology and Applied Pharmacology, Institute of Experimental and Clinical Research, Université Catholique de Louvain, Brussels, Belgium; ^5^Department of Clinical Chemistry, Université Libre de Bruxelles, Brussels, Belgium; ^6^Chest Department, Erasme University Hospital, Université Libre de Bruxelles, Brussels, Belgium; ^7^Department of Cardiology, Centre Hospitalier Universitaire Saint-Pierre, Université Libre de Bruxelles, Brussels, Belgium; ^8^Cardio-Pulmonary Exercise Laboratory, Université Libre de Bruxelles, Brussels, Belgium

**Keywords:** electronic nicotine delivery systems, metabolomics, nicotine, pneumoproteins, transcutaneous oxygen tension

## Abstract

Propylene glycol and glycerol are e-cigarette constituents that facilitate liquid vaporization and nicotine transport. As these small hydrophilic molecules quickly cross the lung epithelium, we hypothesized that short-term cessation of vaping in regular users would completely clear aerosol deposit from the lungs and reverse vaping-induced cardiorespiratory toxicity. We aimed to assess the acute effects of vaping and their reversibility on biological/clinical cardiorespiratory parameters [serum/urine pneumoproteins, hemodynamic parameters, lung-function test and diffusing capacities, transcutaneous gas tensions (primary outcome), and skin microcirculatory blood flow]. Regular e-cigarette users were enrolled in this randomized, investigator-blinded, three-period crossover study. The periods consisted of nicotine-vaping (nicotine-session), nicotine-free vaping (nicotine-free-session), and complete cessation of vaping (stop-session), all maintained for 5 days before the session began. Multiparametric metabolomic analyses were used to verify subjects’ protocol compliance. Biological/clinical cardiorespiratory parameters were assessed at the beginning of each session (baseline) and after acute vaping exposure. Compared with the nicotine- and nicotine-free-sessions, a specific metabolomic signature characterized the stop-session. Baseline serum club cell protein-16 was higher during the stop-session than the other sessions (*P* < 0.01), and heart rate was higher in the nicotine-session (*P* < 0.001). Compared with acute sham-vaping in the stop-session, acute nicotine-vaping (nicotine-session) and acute nicotine-free vaping (nicotine-free-session) slightly decreased skin oxygen tension (*P* < 0.05). In regular e-cigarette-users, short-term vaping cessation seemed to shift baseline urine metabolome and increased serum club cell protein-16 concentration, suggesting a decrease in lung inflammation. Additionally, acute vaping with and without nicotine decreased slightly transcutaneous oxygen tension, likely as a result of lung gas exchanges disturbances.

## INTRODUCTION

Propylene glycol and glycerol, the main constituents of electronic-cigarette (e-cigarette) liquid (e-liquid), produce an aerosol when heated that carries flavoring and nicotine. High-wattage vaping, which enhances heat and aerosol production, is the modality of choice for regular users (vapers) (10–12, 57). High-wattage vaping, with and without nicotine, has been shown to induce transcutaneous hypoxia, constriction of the airways, and lung inflammation in healthy naïve vapers (10, 12). The latter was marked by a rise in serum club cell secretory protein-16 (CC16) without a change in surfactant protein-D (10–12). Acute nicotine-free-vaping decreased partial pressure of arterial oxygen (O_2_) and the oxyhemoglobin fraction in heavy tobacco smokers (naïve vapers), suggesting lung gas exchange disturbances (12).

Propylene glycol and glycerol are small hydrophilic molecules that swiftly cross the lung epithelium (19, 21, 42). When vaped in large amounts, however, this aerosol can transiently accumulate deep in the lungs (42) and interact with the epithelium (50). This hygroscopic and hyperosmolar deposit could theoretically disrupt the rheological properties of surfactant and mucus (21, 23, 31, 49, 50, 52), resulting in bronchiolar and alveolar collapse and therefore impairments to lung gas exchange (40). This possibility is supported by in vitro and animal studies (26, 42, 43, 58, 59), but it is not known if it also occurs in humans (44). We hypothesized that short-term cessation of vaping in regular heavy e-cigarette-users would completely clear aerosol deposit from the lungs, with subsequent recovery of gas exchange and restoration of biological/clinical cardiorespiratory parameters. We also explored whether e-cigarette cessation for 5 days could shift serum and urine metabolomes toward a healthier cardiorespiratory profile. These unanswered questions are important because an increasing proportion of users choose to vape after successfully giving up tobacco smoking (57).

## METHODS

### Participants

Deemed healthy, former tobacco smokers with exclusive nicotine e-cigarette use for at least 1 yr were recruited via a Belgian vaping forum (UBV–BDB–Union-Belge-Pour-La-Vape/Belgische-Damp-Bond). Participants were excluded if they had used chronic medication or recreational drugs. A medical history and physical examination were obtained before enrollment in the study. Healthy participants had the following: *1*) no acute or chronic illness; *2*) no past or present symptoms of cardiopulmonary disease; *3*) no medication use; and *4*) no hypertension as defined by clinical guidelines (57). Participants were also systematically screened for urine tetrahydrocannabinol (semiquantitative detection threshold of 50 ng/mL; NarcoCheck), which had to be negative before each session for inclusion. The study was performed at Erasme University Hospital, Brussels, Belgium between January 2018 and November 2018. Written informed consent was obtained from all the study participants, and the study was approved by the local Ethics Committee (Hôpital-Erasme-CCB-B406201629930) and conformed to the Declaration of Helsinki. The study is registered at ClinicalTrials.gov-identifier-NCT03410511.

### Study Design, Randomization, and Masking

A randomized, investigator-blinded, three-period crossover study was carried out. Allocation to the sequence order was performed by a computer-generated randomization list (Supplemental Fig. S1; all supplemental material is available at https://doi.org/10.5281/zenodo.3478423). A minimum of 7 days separated each period. The periods included: *1*) regular vaping of e-cigarettes containing nicotine for 5 days and until 2 h before the experimental session (nicotine-session); *2*) nicotine-free-vaping for 5 days and until 2 h before the experimental session (nicotine-free-session); and *3*) complete cessation of e-cigarette vaping for 5 days before the experimental session (stop-session).

**Fig. 1.**
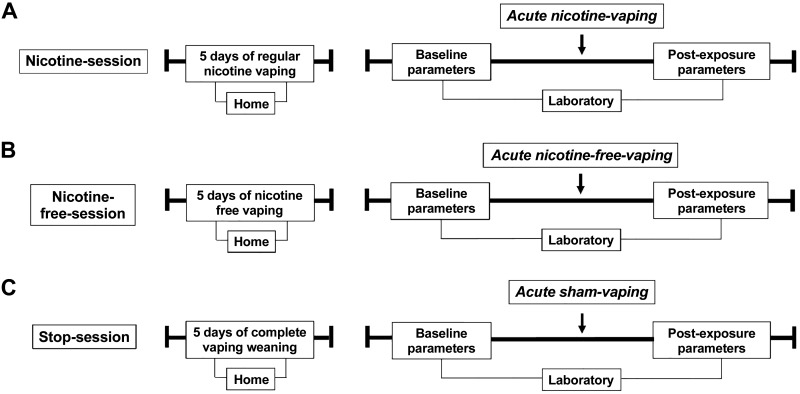
Typical course of the three experimental periods. The periods consisted of regular nicotine e-cigarette vaping for 5 days before the experimental session (nicotine-session) (*A*); nicotine-free-vaping for 5 days before the experimental session (nicotine-free-session) (*B*); and complete cessation of e-cigarette vaping for 5 days before the experimental session (stop-session) (*C*). Biological/clinical cardiorespiratory parameters (serum/urine pneumoproteins, continuous hemodynamic parameters, transcutaneous gas tensions, and skin microcirculatory blood flow) were assessed at the beginning of each session and after acute vaping exposure: 10 nicotine puffs (acute nicotine-vaping in the nicotine-session) (*A*); 10 nicotine-free puffs (acute nicotine-free-vaping in the nicotine-free-session) (*B*); and 10 sham puffs (acute sham-vaping in the stop-session) (*C*).

Baseline blood/urine samples and clinical cardiorespiratory parameters were collected at the beginning of each experimental session. Participants were then invited to vape acutely as follows: *1*) 10 nicotine puffs at 60 W (mean ± SE, 1 ± 0.05 g) (acute nicotine-vaping in the nicotine-session); *2*) 10 nicotine-free puffs at 60 W (1 ± 0.04 g) (acute nicotine-free-vaping in the nicotine-free-session); and *3*) 10 sham puffs (acute sham-vaping in the stop-session) (Fig. 1). The e-liquid base of propylene glycol/glycerol was mixed by the pharmacy at Erasme University Hospital (50:50 vol/vol; pharmaceutical grade; Fagron, Waregem, Belgium). One e-liquid lacked nicotine, and the other contained nicotine at a concentration of 1.5 mg/mL. We used a fourth-generation e-cigarette set at 60 W [Alien 220 box mod, TFV8 baby beast tank and a dual Kanthal coil (V8 Baby-Q2 Core; 0.4Ω dual coils); Smoke, Shenzen, China) with MXJO (Mxjotech, Shenzen, China) IMR 18650 3000 mAh 35A variable voltage/variable wattage batteries. Airflow was set at the maximum. The manufacturer’s recommendations were followed for the preparation of the vaping devices; they were cleaned and filled with e-liquid before each exposure. Batteries were fully charged before use, and the coil was replaced after two exposures. E‐cigarette exposure was carefully controlled: every 30 s, the participant inhaled vaporized aerosol for 3 s, held the aerosol for 2 s, then exhaled. Visual verification of vapor exhalation was used to avoid superficial vaping. This procedure was repeated for 10 puffs. Subjects were asked about symptoms (e.g., cough/throat irritation) following vaping. Sham-vaping was identical to active vaping but with the e-cigarette turned off; this was strictly supervised. The device was weighed before and after each exposure to assess the quantity of e-liquid consumed (10–12). Volunteers were unmasked so they could notice if the vaping device was turned on or off. The investigators were blinded to the experimental session, as both allocation and exposure to the sessions were supervised by an unblinded member who did not participate in any other aspect of the study (10–12). The acute vaping/sham-vaping exposure was followed by blood/urine sampling, clinical cardiorespiratory parameter assessment, lung function tests, and diffusing capacity evaluation.

### Study Assessments

The measurements were carried out with the participant in the semi-supine position after 30 min of rest in a quiet-room maintained at 23 ± 1°C. Speaking and sleeping were not permitted throughout the sessions. The three sessions for each participant commenced at the same time of the day to avoid confounding by nychthemeral variation of cutaneous temperature and club cell protein-16 (CC16) concentration (1, 5). Participants refrained from caffeine, alcohol, and exercise for at least 48 h before each study period. The latter was to avoid the short-term impact of exercise on cutaneous microcirculatory function. Fasting for at least 12 h preceded each experiment; however, water intake was permitted to promote euhydration. Participants were screened for urine tetrahydrocannabinol (semiquantitative detection threshold of 50 ng/mL; NarcoCheck); a positive test resulted in exclusion from the study (10–12).

#### Transcutaneous gas tensions, respiratory parameters, skin microcirculatory blood flow, and hemodynamic parameters throughout the session.

The following parameters were assessed: transcutaneous O_2_ (TcpO_2_) and carbon dioxide (TcpCO_2_) tensions, respiratory parameters [pulse oximetry ($\text{S}{\text{p}}_{{\text{O}}_{2}}$), end-tidal CO_2_, respiratory rhythm], skin microcirculatory blood flow, cutaneous vascular conductance, and hemodynamic parameters [systolic (SBP) and diastolic (DBP) blood pressure, heart rate].

A PeriFlux system 5000 (Perimed, Järfälla, Sweden) was used to monitor TcpO_2_ and TcpCO_2_ tensions by means of a PF 5040 unit and a dual TcpO_2_/CO_2_ E5280 electrode. The E5280 electrode, covered by a membrane permeable to oxygen (O_2_) and carbon dioxide (CO_2_) heated (44°C) the underlying tissue to maximize gas diffusion through the skin. Direct polarography was used to compute TcpO_2_ tension. The electrode, which consists of a silver anode and a platinum cathode, generates a current when the O_2_ is reduced. This current is subsequently converted into voltage and digitalized. TcpCO_2_ tension was measured electrochemically through a change in pH of the electrolyte solution located between the electrode and the membrane. A TC 550 fixation ring (Perimed, Järfälla, Sweden) was applied to the skin on the anterior side of the right forearm (lower third, 5 cm distal to the antecubital fossa) at the level of the heart after the area was wiped with ethyl alcohol. Four drops of contact liquid (TC 560, Perimed, Järfälla, Sweden) were instilled on the inside of the TC 550 fixation ring. The electrode was placed, avoiding superficial vessels and body hair, at the same location for each of the three sessions. This was based on multiple images taken during the first experimental session. Calibration was carried out before each session as recommended by the manufacturer. When variations did not exceed ± 2 mmHg within 1 min, TcpO_2_/CO_2_ values were considered stable. Every two sessions, electrode re-membraning was performed (45, 53, 65).

Continuous monitoring of $\text{S}{\text{p}}_{{\text{O}}_{2}}$, end-tidal carbon dioxide pressure, respiration rhythm, and heart rate were assessed with the Capnostream-35-monitor (Oridion Medical, Jerusalem, Israël).

A PeriFlow system 5000, PF 5010/5020 with the thermostatic probe 457 (Perimed, Järfälla, Sweden) at least 5 cm from the PF 5040 TcpO_2_/TcpCO_2_ unit, was used to assess skin microcirculatory blood flow. The thermostatic probe 457 emits a laser beam that penetrates skin tissue. A change in wavelength occurs by Doppler shift when the beam encounters a moving cell within cutaneous vessels. The probe was heated to 33°C during the study sessions to avoid modifications in microcirculatory blood flow induced by cutaneous temperature changes (62). Cutaneous vascular conductance was calculated by dividing skin microcirculatory blood flow by mean arterial pressure obtained by a Finometer device (15). SBP and DBP waveforms were obtained throughout the sessions from the right middle finger using Finometer Pro (FMS, Amsterdam, The Netherlands) (27).

Humeral blood pressure was determined after 30 min of rest in the supine position (Omron, Kyoto, Japan) according to guidelines (66). Blood pressure was taken before (baseline) and immediately following vaping or sham-vaping sessions. A cuff was placed on the middle phalanx of the right middle finger to obtain a finger blood pressure waveform with a beat-to-beat hemodynamic monitoring system (Finometer Pro, FMS, Amsterdam, The Netherlands). Humeral blood pressure waveforms were reconstructed simultaneously via a generalized transfer function. Thus, continuous monitoring of the humeral SBP and DBP throughout all the experimental sessions was enabled. Finometer recordings were used to monitor pulse rates throughout the study (27).

#### Lung function and diffusing capacity.

Lung function tests were performed in accordance with guidelines 3 h after acute vaping exposure using Hyp’Air-Compact+ (Medisoft, Sorinnes-Dinant, Belgium). Flow volume curves (forced expiration and inspiration), body plethysmography (lung volumes), and measurements of lung diffusing capacities for carbon monoxide (DLCO) and nitric oxide (DLNO) measured with a double NO/CO single breath technique were obtained (2, 18).

#### Blood and urine samples at baseline and after acute exposure.

Blood was drawn from the left antecubital at the beginning of the session (baseline) and 60 min after acute vaping or sham-vaping. After being drawn, blood was immediately centrifugated at 3,500 *g* for 10 min to obtain the supernatant. This was then aliquoted. Urine specimens were collected at the beginning of the session and 90 min after acute exposure. Immediately after centrifugation and aliquoting, serum, plasma, and urine samples were frozen and stored at −80°C.

##### pneumoproteins, creatinine, and retinol binding protein.

Latex immunoassay using a rabbit anti-CC16-antibody (Dakopatts, Glostrup, Denmark) was used to measure CC16 in serum and urine (63), while standard CC16 was assessed at the Louvain Centre for Toxicology and Applied Pharmacology, Faculty of Medicine, Catholic University of Louvain. Urine retinol binding protein and urine and serum creatinine were quantified by the Beckman-Synchron-CX5-Delta-Clinical System (Beckman Coulter, Fullerton, CA) (63). Concentrations of serum and urine CC16 were adjusted for serum and urine creatinine, respectively. A commercially available ELISA kit (Biovendor, Karasek, Czech Republic) was used to determine the serum concentration of surfactant protein-D (SP-D), as previously described (63).

##### measures to ensure strict compliance of the study protocol: urine cotinine, exhaled carbon monoxide, serum nicotine, and propylene glycol.

Each participant signed a declaration at the beginning of each experimental session confirming adherence to the study protocol. Serum nicotine and propylene glycol at baseline and urine cotinine (semiquantitative detection threshold of 600 ng/mL; NarcoCheck, Paris, France) and fractional concentration of carbon monoxide (CO) during expiration were also measured to ensure strict compliance to the protocol.

Serum nicotine was assessed before and 60 min after the nicotine-vaping by means of a mass spectrometer (Agilent QQQ 6490, Agilent, Santa Clara, CA) with a jet stream electrospray ion source, as previously described (11).

Serum propylene glycol was assessed using gas chromatography with a flame ionization detector (Agilent; ref: GC HP 6890-FID) following precipitation with acetonitrile (Biosolve; ref: UN1648), as previously described (12, 46).

##### serum and urine metabolomics analysis.

Urine samples were centrifuged at 1,600 *g* for 5 min at 4°C. Then, 500 μL of urine were mixed with 250 μL of phosphate buffer (0.04 M Na_2_HPO_4_, 0.2 M NaH_2_PO_4_, pH 7.4) and prepared in H_2_O/D_2_O (80:20; vol/vol) to minimize pH variation (PB). The samples were then centrifuged at 13,000 *g* for 10 min. Fifty microliters of a 14 mM solution of 3-TrimethylSilyl Propionic-2,2,3,3-d4 acid solution prepared in 100% deuterium oxide (TSP) were added to 650 µL of the supernatant. Of this mixture, 700 μL were then transferred into 5-mm NMR tubes, and ^1^H-NMR spectra were recorded at 300° K using a Bruker Avance 500 spectrometer working at 11.8 T. One-dimensional spectroscopy was performed using a NOESYPRESAT-1D pulse sequence, 64 scans, 54,832 data points, a spectral width of 10,330.578 Hz, an acquisition time of 2.65 s, and a pulse recycle delay of 3 s. FIDs were Fourier transformed, and a line broadening of 0.3 Hz was applied (54).

Serum samples (250 μL) were mixed with 500 μL of PB and centrifuged at 13,000 *g* for 10 min. Then, 50 μL of a 14 mM solution of TSP were added to 650 μL of the supernatant. Of this mixture, 700 μL were then transferred into 5-mm NMR tubes, and ^1^H-NMR spectra were recorded at 300°K using a Bruker Avance 500 spectrometer working at 11.8 T. One-dimensional spectroscopy was performed using a CPMGPR1D pulse sequence, 128 scans, 54,832 data points, a spectral width of 10,330,578 Hz, an acquisition time of 1.99 s, and a pulse recycle delay of 1 s. FIDs were Fourier transformed, and a line broadening of 0.3 Hz was applied (60).

### Data Management

Data analysis was carried out blindly. The details regarding missing data are provided in Supplemental Tables S.1.1, S.1.2, and S.1.3.

Transcutaneous gas (O_2_ and CO_2_) readings were recorded and analyzed offline (Perisoft 2.5.5, Perimed, Järfälla, Sweden). Thirty minutes after sensor fixation, TcpO_2_ and TcpCO_2_ recordings began and continued throughout the exposure session. The mean of the recordings taken in the 5-min interval from 30–35 min after the sensor was fixed in place represented the baseline values. Recording of baseline values occurred immediately before vaping or sham-vaping. Recordings taken over the full duration of e-cigarette exposure were recorded as a single average value. Transcutaneous gas parameters were then followed over the next 60 min; these were grouped and averaged over 6 successive 10-min intervals.

The Capnostream 35 (Oridion Medical, Jerusalem, Israel) was used to continuously monitor $\text{S}{\text{p}}_{{\text{O}}_{2}}$, end-tidal carbon dioxide, respiratory rhythm, and heart rate. Baseline values were obtained as the mean of 5-min recordings taken from 30–35 min after sensor fixation. Recording of baseline values immediately preceded vaping or sham-vaping exposure. Recordings obtained during the entire period of e-cigarette exposure were averaged to a single value. Thereafter, we followed these parameters over the next 60 min, which were grouped and averaged over 6 successive 10-min intervals.

Skin microcirculatory blood flow readings were recorded and analyzed offline (Perisoft 2.5.5, Perimed, Järfälla, Sweden). Skin microcirculatory blood flow recordings began 30 min after sensor fixation and continued for the duration of the session. The mean of the recordings taken in the 5-min interval from 30–35 min after sensor fixation represented the baseline values. Recording of baseline values occurred immediately before vaping or sham-vaping. Recordings taken over the full duration of e-cigarette exposure were recorded as a single average value. Skin microcirculatory blood flow was then followed over the next 60 min; these measurements were grouped and averaged over 6 successive, 10-min intervals.

Hemodynamic measurements were taken using the Finometer Pro. Finometer values were recorded and analyzed offline (Beatscope 1.1a, FMS, Amsterdam, The Netherlands). The baseline period and vaping exposure were summarized using one value. We subsequently recorded hemodynamic parameters for 60 min. Averages were taken of 6 successive periods of continuous recording lasting 10 min each. Finometer hemodynamic values were adjusted for humeral blood pressure values.

For serum and urine metabolomic data management, the spectra were automatically phase- and baseline-corrected using MestReNova 10.0 software (Mestrelab Research, Santiago de Compostela, Spain). Spectral chemical shifts were calibrated according to the resonance of TSP and arbitrarily fixed at 0.00 parts per million (ppm), and all peak intensities were normalized to the reference, whose intensity was arbitrarily fixed at a value of 100. The spectral region ranging from 0.08–10.00 ppm was binned 0.04-ppm stepwise into 248 subregions. The regions ranging from 4.50–6 ppm were excluded from the analysis to remove the residual water signal and the contribution of the urea resonance that may fluctuate due to daily variations. Each integrated region was normalized to the total spectrum area to compensate for possible variations in urine dilution/concentration effects among the samples. The final data set was imported into SIMCA-P+12.0 software (Umetrics, Umea, Sweden). After mean-centering of the data, a partial least squares discriminant analysis (PLS-DA) was performed. The obtained scores’ scatter plots and their corresponding loading scatter plots facilitated the global view on the relationships between observations and the determination of spectral area possibly differing between sessions. The variables of importance in the projection (VIP) lists were established. Using a cutoff value of VIP ≥ 1, discriminant metabolites were pinpointed for each session. The VIP ≥ 1 metabolites were then identified using in-house references, ChenomX (Version 8.1, ChenomX, Canada), and the Human Metabolome DataBase and classed in a heatmap plot using Morpheus (https://software.broadinstitute.org/morpheus) (42).

### Outcome Measures

The primary outcome was the impact of vaping on TcpO_2_. Secondary outcomes included TcpCO_2_, continuous respiratory parameters, skin microcirculatory blood flow, cutaneous vascular conductance, hemodynamic parameters, lung function/diffusing capacity evaluation, serum/urine pneumoproteins, and baseline serum/urine metabolomes.

### Statistical Analysis

After internal discussion, we reported in the trial registration an anticipated enrollment of at least 21 participants, who had to perform the 3 experimental sessions.

Continuous data were tested for normality using the Kolmogorov–Smirnov test. The mean ± SE is reported if data were normally distributed or otherwise median [interquartile range, P25–P75]. An intention-to-treat analysis was performed. The baseline data were compared between the three sessions, and the differences between baseline and the post-acute exposure value (Δ) were compared to assess the effects of acute nicotine- and nicotine-free-vaping compared with acute sham-vaping. Mixed linear models were used with experimental sessions and time points as fixed effects and subjects’ baselines as random effects (random-intercept-model) variables. Potential autocorrelation and heteroscedasticity were included in the model. The Bonferroni–Holm method was used to counteract the problem of multiple comparisons. Correlation analyses used the Pearson’s correlation coefficient. The R-software was used, with statistical significance set at 0.05 (two-tailed) (13). Multivariate data analyses (PLS-DA) were performed on urine and serum metabolomic final data set. A false discovery rate procedure correction was not conducted in the metabolomic study. Discriminant metabolites (variables of importance) were pinpointed for each session, identified, and classed in a heatmap plot using Morpheus (42).

## RESULTS

**Fig. 2.**
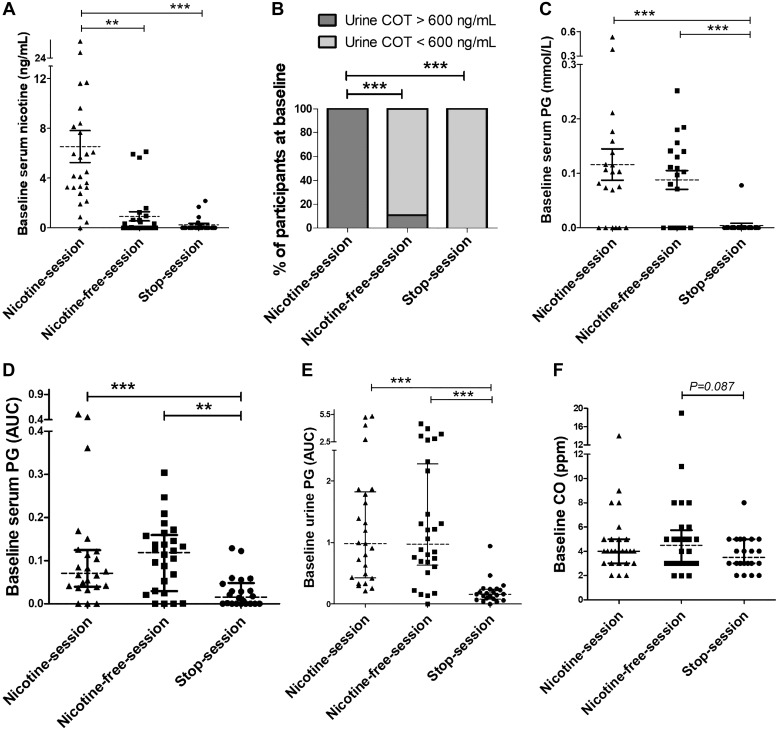
Baseline values of serum nicotine (*A*), semiquantitative assessment of urine cotinine (COT) (*B*), serum propylene glycol (PG) assessed by gas chromatography with a flame ionization detector (*C*), area under the curve (AUC) of serum PG assessed by ^1^H-NMR spectroscopy (*D*), AUC of urine PG (^1^H-NMR spectroscopy) (*E*), and exhaled carbon monoxide (CO) (*F*). Horizontal brackets represent the *P* values for the comparison between baseline values in each session. A mixed-effects linear model analysis was performed with experimental sessions and time points as fixed effects and baseline values as random effects (random intercept model). **P < 0.01, ***P < 0.001. Data represent the mean ± SE.

The study included 30 male participants with a mean age of 38 ± 2 yr. They were regular and exclusive e-cigarettes users since 38 ± 3 mo, with a mean daily e-liquid consumption of 17 ± 2 mL (Table 1). Whereas the baseline level of serum nicotine and urine cotinine were in accordance with the study protocol for 72 (95%) study sessions, abnormally high levels of baseline serum nicotine (>2 ng/mL) (20) and urine cotinine (>600 ng/mL) (32) were found in 3 nicotine-free-sessions and in 1 stop-session (further details in Table 2 and Fig. 2).

**Table 1.** Baseline clinical and usual consumption trends of the study participants

	Participants (*n* = 30)
Age, yr	38 ± 2
Size, cm	181 ± 1
Weight, kg	88 ± 4
BMI, kg/m^2^	26 ± 1
Past history of tobacco smoking (pack-years)	18 ± 2
Years of tobacco abstinence, yr	3.1 ± 0.3
E-cigarette consumption, month	38 ± 3
Direct inhale, %	83 ± 7
Usual e-liquid nicotine strength, mg/mL	2.9 ± 0.3
Usual e-liquid consumption per day, mL	17 ± 2
Usual e-liquid PG/GLY ratio, %	60 ± 8
Usual e-cigarette resistance, Ω	0.3 ± 0.03
Usual e-cigarette power, W	53 ± 5

BMI, body mass index; e-cigarette, electronic cigarette; PG, propylene glycol; GLY, glycerol.

**Table 2.** Baseline parameters according to nicotine-session, nicotine-free-session, and stop-session

	Nicotine-Session^1^	Nicotine-Free-Session^2^	Stop-Session^3^	*P*^1 vs. 3^	*P*^2 vs. 3^	*P*^1 vs. 2^
E-cigarette use biomarkers						
Serum propylene glycol by GC-FID, mmol/L†	0.1 [0–0.15]	0.09 [0–0.14]	0 [0–0]	<0.001	<0.001	0.19
Serum propylene glycol by ^1^H-NMR, AUC	0.07 [0.04–0.12]	0.12 [0.03–0.16]	0.01 [0–0.05]	0.003	0.008	0.752
Urine propylene glycol by ^1^H-NMR, AUC†	1 [0.4–1.8]	0.9 [0.6–2.2]	0.2 [0.1–0.2]	<0.001	<0.001	0.698
Serum nicotine, ng/mL†	3.9 [1.7–8.2]	0 [0–0.7]	0 [0–0]	0.001	0.366	<0.001
Urine cotinine > 600 ng/mL, %‡	100	11	0	0.001	0.246	<0.001
Fraction exhaled carbon monoxide	4 [3–5]	4.5 [3–5.8]	3.5 [3–5]	0.217	0.087	0.622
Respiratory parameters						
Peripheral pulse oximetry, %*	96.3 ± 0.4	96.2 ± 0.4	95.7 ± 0.5	0.006	0.016	0.672
Transcutaneous oxygen tension, mmHg*	76.1 ± 1.9	76 ± 1.7	75.3 ± 2.2	0.435	0.37	0.915
Transcutaneous carbon dioxide tension, mmHg*	37.1 ± 0.5	37.2 ± 0.5	37.9 ± 0.6	0.109	0.255	0.606
End-tidal carbon dioxide, mmHg*	37.7 ± 0.4	38.1 ± 0.5	37.7 ± 0.7	0.633	0.807	0.446
Respiratory rate, min^−1^*	15 ± 1	15 ± 1	15 ± 1	0.628	0.647	0.97
Hemodynamic parameters						
Heart rate, beats/min*	66 ± 2	63 ± 1	63 ± 2	<0.001	0.851	<0.001
Humeral systolic blood pressure, mmHg*	117 ± 3	116 ± 3	115 ± 3	0.497	0.602	0.863
Humeral diastolic blood pressure, mmHg*	79 ± 2	78 ± 2	77 ± 2	0.242	0.545	0.547
Cutaneous blood flow						
Skin microcirculatory blood flow, PU*	14 ± 2	13 ± 1	12 ± 1	0.958	0.55	0.496
Cutaneous vascular conductance, PU/mmHg*	0.15 ± 0.02	0.15 ± 0.01	0.14 ± 0.01	0.8	0.515	0.345
Pneumoproteins						
Serum CC16, µg/L†	7.6 [6.1–9.4]	6.5 [5.2–9.4]	8.1 [6.8–10.3]	0.011	0.004	0.685
Serum SP-D, µg/L†	97 [69–140]	107 [81–146]	105 [73–138]	0.768	0.164	0.252
Urine CC16/urine creatinine, ng/mg†	12.6 [9–26.6]	18.1 [10–32.2]	16.4 [7.7–36.1]	0.062	0.745	0.103

^1^ H-NMR, nuclear magnetic resonance spectroscopy; AUC, area under the curve; CC16, club cell protein-16; GC-FID, gas chromatography with a flame ionization detector; PU, perfusion unit (arbitrary unit); SP-D, surfactant protein-D.

* The data are the mean ± SE;

† The data are the median [interquartile range];

‡ Categorical variables were studied using the chi-square test.

### Transcutaneous Gas Tensions, Respiratory Parameters, and Skin Microcirculatory Blood Flow Throughout the Session

#### Effect of vaping cessation on baseline values.

**Fig. 3.**
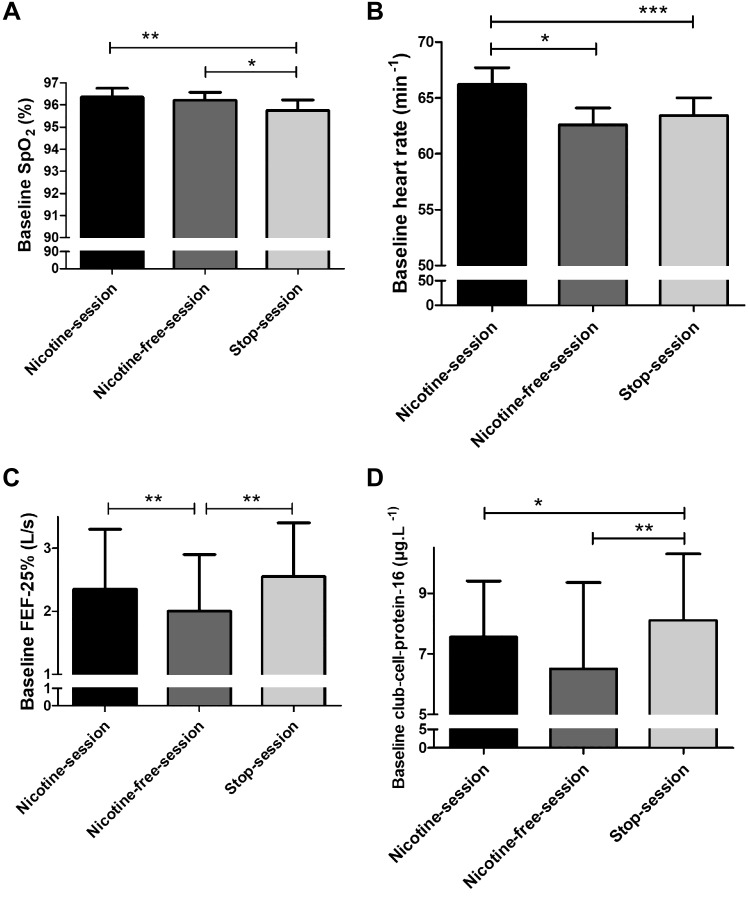
Baseline values of peripheral pulse oximetry ($\text{S}{\text{p}}_{{\text{O}}_{2}}$) (*A*), heart rate (*B*), forced expiratory flow at 25% (FEF-25%) (*C*), and club cell protein-16 (*D*). Horizontal brackets represent the *P* values for the comparison between baseline values in each session. A mixed-effects linear model analysis was performed with experimental sessions and time points as fixed effects and baseline values as random effects (random intercept model). *P < 0.05, **P < 0.01, ***P < 0.001. Data are presented as the mean ± SE (*A* and *B*) or the median [interquartile range, P25–P75] (*C* and *D*).

The $\text{S}{\text{p}}_{{\text{O}}_{2}}$ baseline value was lower in the stop-session (95.7 ± 0.5%) compared with the nicotine-session (96.3 ± 0.4%; *P* = 0.006) and the nicotine-free-session (96.2 ± 0.4%; *P* = 0.016; Fig. 3*A*). In contrast, baseline values of TcpO_2_, TcpCO_2_, end-tidal CO_2_, respiratory rhythm, skin microcirculatory blood flow, and cutaneous vascular conductance did not change (Table 2).

#### Acute vaping exposure.

**Fig. 4.**
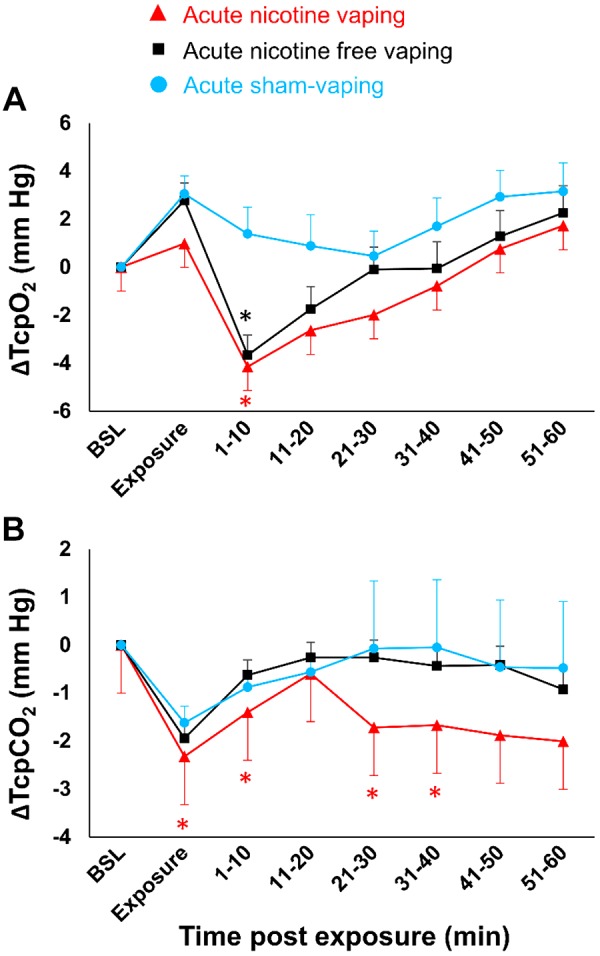
Absolute changes over time in transcutaneous partial pressures of oxygen (TcpO_2_) (*A*) and carbon dioxide (TcpCO_2_) (*B*) after acute nicotine-vaping (nicotine-session) (red triangles), acute nicotine-free-vaping (nicotine-free-session) (black squares), and acute sham-vaping (stop-session) (blue dots). Compared with acute sham-vaping, acute nicotine- and nicotine-free-vaping were associated with slight decreases in TcpO_2_ for 10 min postexposure. Compared with acute sham-vaping, acute nicotine-vaping was associated with significant decreases in TcpCO_2_ that persisted for 40 min postexposure. The red asterisks represent significant *P* values from the comparison of acute nicotine-vaping vs. acute sham-vaping, and the black asterisks represent significant *P* values from the comparison of acute nicotine-free-vaping vs. acute sham-vaping. A mixed-effects linear model analysis was performed with experimental sessions and time points as fixed effects and baseline (BSL) values as random effects (random intercept model). *P < 0.05. Data are the mean ± SE.

**Fig. 5.**
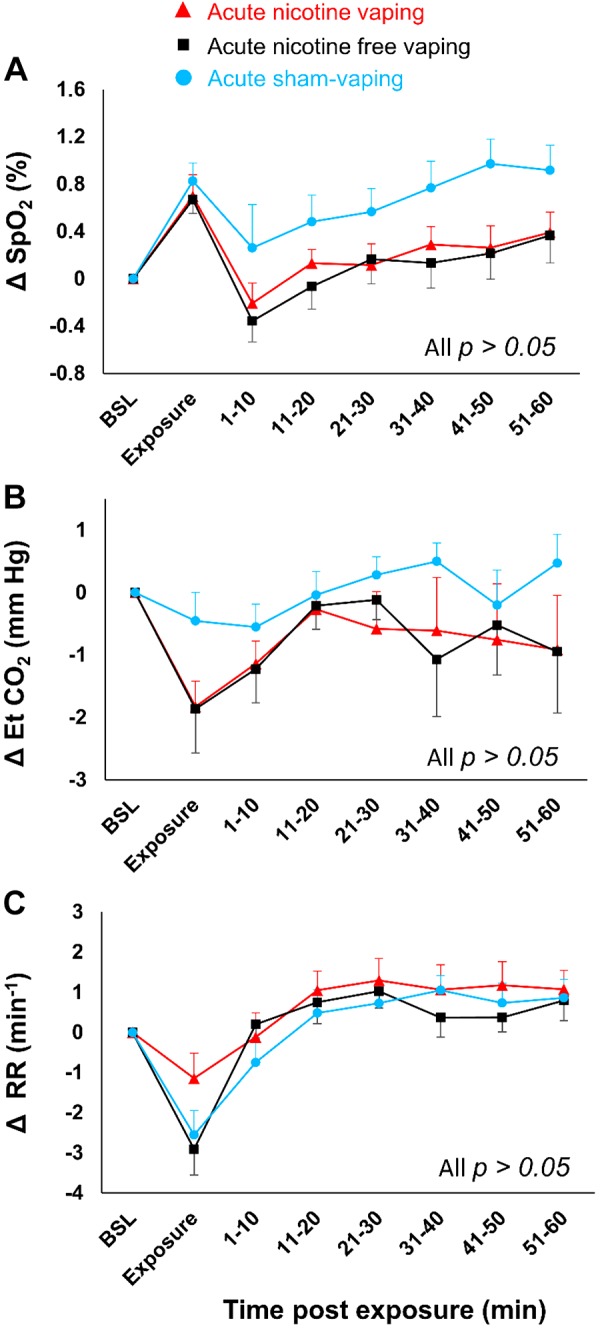
Absolute changes over time in saturation of hemoglobin with oxygen as measured by pulse oximetry oxygen ($\text{S}{\text{p}}_{{\text{O}}_{2}}$) (*A*), end-tidal carbon dioxide (EtCO_2_) (*B*), and respiratory rhythm (RR) (*C*) in case of acute nicotine-vaping (nicotine-session) (red triangles), acute nicotine-free-vaping (nicotine-free-session) (black squares), and acute sham-vaping (stop-session) (blue dots). In comparison to acute sham-vaping, neither acute nicotine-vaping nor nicotine-free-vaping modified $\text{S}{\text{p}}_{{\text{O}}_{2}}$, EtCO_2_, or RR. A mixed-effects linear model analysis was performed with experimental sessions and time points as fixed effects and baseline (BSL) variables as random effects (random intercept model). Data are presented as the mean ± SE.

**Fig. 6.**
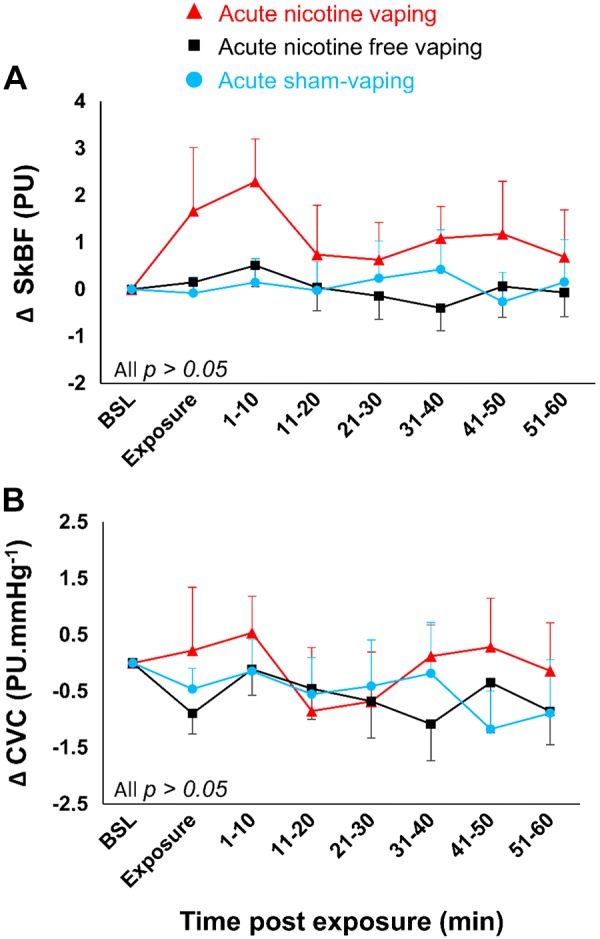
Absolute changes over time in skin microcirculatory blood flow (SkBF) (*A*) and cutaneous vascular conductance (CVC) (*B*) in case of acute nicotine-vaping (nicotine-session) (red triangles), acute nicotine-free-vaping (nicotine-free-session) (black squares), and acute sham-vaping (stop-session) (blue dots). In comparison to acute sham-vaping, neither acute nicotine-vaping nor acute nicotine-free-vaping modified SkBF and CVC. A mixed-effects linear model analysis was performed with experimental sessions and time points as fixed effects and baseline (BSL)variables as random effects (random intercept model). Data are presented as the mean ± SE. PU, perfusion unit (arbitrary unit).

Δ-TcpO_2_ decreased 10 min after acute nicotine-vaping (−4.1 ± 1.1 vs. +1.4 ± 0.8 mmHg; *P* = 0.016) and acute nicotine-free-vaping (−3.7 ± 0.8 vs. +1.4 ± 0.8 mmHg, *P* = 0.039) compared with acute sham-vaping (Fig. 4*A*). Additionally, Δ-TcpCO_2_ decreased 40 min after nicotine-vaping (−1.7 ± 1.4 vs. −0.05 ± 0.32 mmHg; *P* = 0.045; Fig. 4*B*) compared with acute sham-vaping. Acute nicotine-vaping, nicotine-free-vaping, and sham-vaping resulted in similar changes in terms of Δ-$\text{S}{\text{p}}_{{\text{O}}_{2}}$, Δ-end-tidal CO_2_, Δ-respiratory rhythm, Δ-skin microcirculatory blood flow, and Δ-cutaneous vascular conductance (Figs. 5 and 6). Positive correlations were found between Δ-$\text{S}{\text{p}}_{{\text{O}}_{2}}$and Δ-TcpO_2_ after acute nicotine-vaping (*r* = 0.610; *P* = 0.004) and acute nicotine-free-vaping (*r* = 0.687; *P* < 0.001).

### Hemodynamic Parameters Throughout the Session

#### Effect of vaping cessation on baseline values.

Baseline heart rate values were higher in the nicotine-session (66 ± 2 beats/min) compared with the nicotine-free-session (63 ± 1 beats/min; *P* < 0.001) and stop-session (63 ± 2 beats/min; *P* < 0.001; Table 2 and Fig. 3*B*).

#### Acute vaping exposure.

**Fig. 7.**
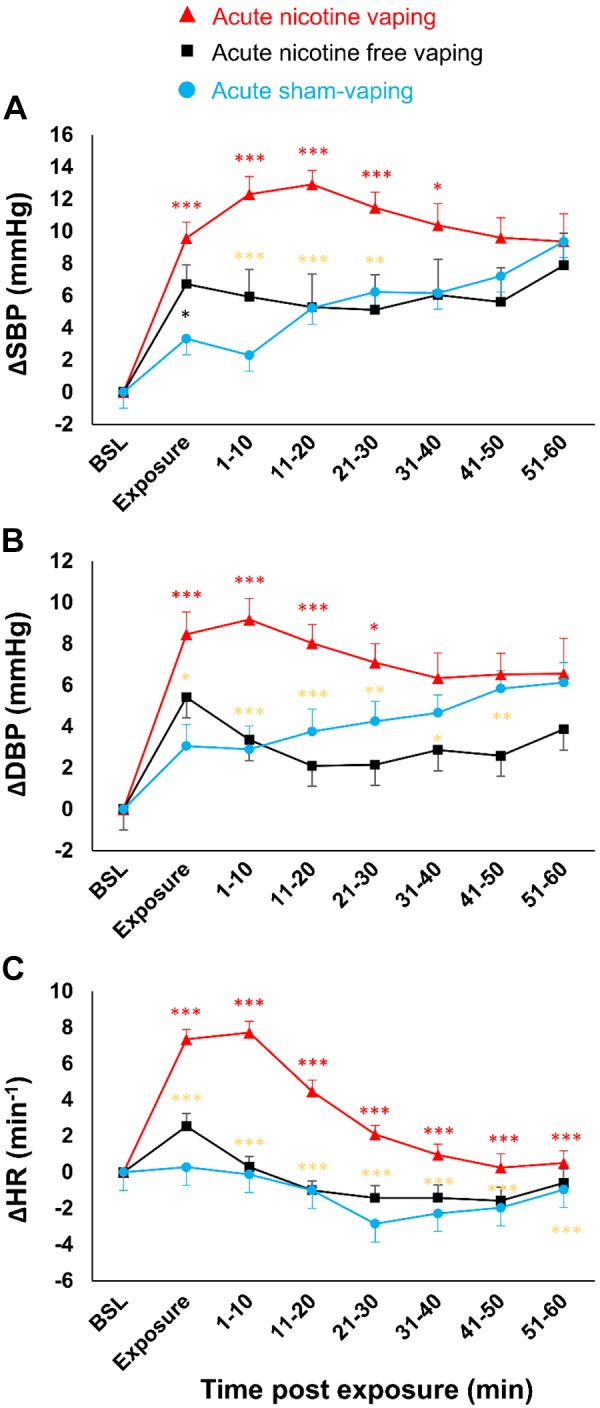
Absolute changes over time in systolic blood pressure (SBP) (*A*), diastolic blood pressure (DBP) (*B*), and heart rate (HR) (*C*) after acute nicotine-vaping (nicotine-session) (red triangles), acute nicotine-free-vaping (nicotine-free-session) (black squares), and acute sham-vaping (stop-session) (blue dots). In comparison to acute sham-vaping, acute nicotine-vaping increased SBP, DBP, and HR for 40, 30, and 60 min postexposure, respectively. In comparison to acute nicotine-free-vaping, acute nicotine-vaping increased SBP, DBP, and HR for 30, 30, and 60 min postexposure, respectively. The red asterisks represent significant *P* values from the comparison of acute nicotine-vaping vs. acute sham-vaping, the black asterisks represent significant *P* values from the comparison of acute nicotine-free-vaping vs. acute sham-vaping, and the gold asterisks represent significant *P* values from the comparison of acute nicotine- and nicotine-free-vaping. A mixed-effects linear model analysis was performed with experimental sessions and time points as fixed effects and baseline (BSL) values as random effects (random intercept model). *P < 0.05, **P < 0.01, ***P < 0.001. Data represent the mean ± SE.

In comparison to acute sham-vaping, 20 min after acute nicotine-vaping, Δ-SBP increased from 5 ± 1 to 13 ± 2 mmHg (*P* < 0.001), Δ-DBP increased from 4 ± 1 to 8 ± 1 mmHg (*P* < 0.001), and Δ-heart rate increased from −1 ± 1 to 4 ± 1 beats/min (*P* < 0.001). Compared with acute nicotine-free-vaping, acute nicotine-vaping increased Δ-SBP from 5 ± 2 to 11 ± 1 mmHg (*P* < 0.001), Δ-DBP from 3 ± 1 to 7 ± 1 mmHg (*P* = 0.007), and Δ-heart rate from −1 ± 1 to 1 ± 1 beats/min (*P* < 0.0001), 30-, 50-, and 60-min postexposure, respectively (Fig. 7).

### Lung Function Tests and Diffusing Capacity After Acute Exposure

Forced expiratory flow-25% (FEF-25%) was higher in the stop-session compared with the nicotine-free-session (2.5 [0.7–3.2] vs. 2.0 [0.6–2.7] L/s; *P* = 0.001; Fig. 3*C*). All other parameters of lung function and diffusing capacities were not modified by any of the three experimental sessions (Table 3).

**Table 3.** Functional pulmonary variables (flow-volume curves and diffusion capacity of nitric oxide and carbon monoxide) in the study population according to the experimental sessions

	Nicotine-Session^1^		Nicotine-Free-Session^2^		Stop-Session^3^				
	Value	% Pred	Value	% Pred	Value	% Pred	*P*^1 vs. 3^	*P*^2 vs. 3^	*P*^1 vs. 2^
Spirometry									
FEV 1 s, L	4.3 [3.6–4.8]	106 [87–112]	4.3 [3.5–5]	104 [88–110]	4.3 [3.5–4.7]	106 [89–112]	0.37	0.512	0.779
FEV 1 s/FVC, %	79 [72–85]	98 [92–104]	78 [73–81]	97 [90–100]	79 [73–83]	100 [90–102]	0.616	0.19	0.061
PEF, L/s	8.8 [7.2–9.8]	92 [78–104]	8.9 [6.4–10.2]	90 [69–105]	7.8 [6.9–9.9]	84 [68–101]	0.398	0.433	0.931
FEF-75%, L/s	8.2 [6–9]	97 [72–106]	8.2 [5.5–9.7]	98 [68–115]	6.9 [5.6–9.5]	85 [67–113]	0.353	0.222	0.777
FEF-50%, L/s	5.3 [4.1–6.5]	95 [75–127]	5 [3.7–6.4]	96 [76–118]	5.5 [4.3–6.4]	104 [83–124]	0.395	0.129	0.494
FEF-25%, L/s	2.3 [1.7–3]	99 [72–117]	2 [1.6–2.8]	85 [69–107]	2.5 [1.9–3.3]	102 [79–135]	0.535	0.001	0.002
Lung diffusing capacity									
DLCO_cor_, mL·min^−1^·mmHg^−1^	30.4 [26.9–38.5]	77 [70–83]	33.9 [29.1–36.6]	76 [62–83]	31.7 [27.2–38.3]	73 [66–83]	0.214	0.182	0.971
DLNO, mL·min^−1^·mmHg^−1^	165.3 [143.4–185.9]	85 [80–96]	165.5 [149–178.1]	84 [67–93]	156.4 [146.8–185.1]	86 [79–99]	0.403	0.284	0.849
Dm_co_, mL/min^−1^·mmHg^−1^	83.9 [72.8–94.7]	86 [80–97]	84 [76.2–91.6]	83 [17–93]	79.2 [72.5–92]	84 [78–96]	0.538	0.143	0.411
Vc_cor_, mL	85.1 [71.8–99]	71 [64–81]	87 [77.4–96.1]	69 [15–77]	82.7 [72.6–95.8]	71 [64–81]	0.46	0.976	0.413
VA, L	7.4 [6.9–8.2]	101 [96–110]	7.4 [6.7–8.3]	98 [24–103]	7.5 [6.9–8.3]	102 [100–109]	0.995	0.487	0.482
DLNO/DLCO_cor_	5.1 [4.8–5.5]	–	5.1 [4.6–5.5]	–	4.8 [4.7–5.3]	–	0.924	0.559	0.49

Data are the median [interquartile range]. DLCO_cor_, diffusing capacities for carbon monoxide corrected for hemoglobin concentration; DLNO, lung transfer capacity for nitric oxide; Dm_co_, membrane diffusion factor; FEF, forced expiratory flow; FEV 1 s, forced expiratory volume in 1 s; FVC, forced vital capacity; PEF, peak expiratory flow; VA, alveolar volume; VC_cor_, lung capillary blood volume corrected for hemoglobin concentration.

### Lung Injury Biomarkers Before and After Exposure

#### Effect of vaping cessation on baseline values.

The baseline serum CC16 concentration was higher in the stop-session (8.1 [6.8–10.3] µg/L) compared with the nicotine-session (7.6 [6.1–9.4] µg/L; *P* = 0.011) and the nicotine-free-session (6.5 [5.2–9.4] µg/L; *P* = 0.004; Table 2; Fig. 3*D*).

#### Effect of acute vaping exposure.

During the nicotine-session, serum nicotine increased after acute nicotine-vaping compared with its baseline (5.5 [4.3–7.3] vs. 3.9 [2–8]; *P* = 0.025, respectively). Δ-serum/urine creatinine, Δ-serum/urine CC16, Δ-serum surfactant protein-D, and Δ-urine retinol-binding-protein did not vary after acute nicotine- and nicotine-free vaping compared with acute sham-vaping (Table 4).

**Table 4.** Serum and urine pneumoprotein concentrations according to the experimental sessions

	Nicotine-Session^1^		Nicotine-Free-Session^2^		Stop-Session^3^				
	BSL	T60MIN	BSL	T60MIN	BSL	T60MIN	*P* ^1 vs. 3^	*P* ^2 vs. 3^	*P* ^1 vs. 2^
Serum creatinine, mg/dL	0.99 [0.9–1.1]	0.95 [0.83–1.02]	0.94 [0.88–1.03]	0.93 [0.84–1.01]	1 [0.86–1.09]	0.91 [0.81–1.08]	0.823	0.827	0.992
Serum CC16, µg/L	7.6 [6.2–9.4]	6.3 [5.4–8.6]	6.5 [5.2–9.4]	6.8 [4.8–8.4]	8.1 [6.8–10.3]	6.1 [5.3–8.1]	0.979	0.585	0.588
Serum CC16/serum creatinine	7.3 [6–9.4]	7.6 [5.1–8.8]	6.7 [5.2–9.6]	7.3 [5.5–8.6]	8.8 [6.9–10.2]	6.5 [5–9.4]	0.907	0.725	0.624
Serum SP-D, µg/L	97 [69–140]	92 [61–141]	107 [81–146]	102 [73–151]	105 [73–138]	97 [67–139]	0.881	0.65	0.529
Urine creatinine, g/L	2.1 [1.3–2.8]	1.5 [1–1.9]	1.6 [1–2]	1.1 [0.8–1.7]	1.8 [1.1–2.2]	1.3 [1–2]	0.427	0.099	0.363
Urine CC16, ng/mL	30.6 [14.7–41.7]	22.4 [15.1–38.5]	27.9 [13.5–46.2]	21.3 [14.6–33.3]	20 [17.4–42.5]	20 [13.2–38.2]	0.885	0.679	0.775
Urine RBP, µg/L	189 [107–307]	155 [73–255]	159 [56–253]	136 [53–221]	196 [84–253]	158 [97–218]	0.345	0.021	0.151
Urine CC16/urine creatinine	12.6 [9–26.6]	17.2 [12.3–27.2]	18.1 [10–32.2]	20.2 [10.5–30.9]	16.4 [7.7–36.1]	16.8 [8.1–30.2]	0.994	0.519	0.491
Urine CC16/urine RBP	0.12 [0.09–0.25]	0.14 [0.08–0.35]	0.16 [0.1–0.45]	0.14 [0.1–0.56]	0.15 [0.09–0.34]	0.13 [0.08–0.27]	0.639	0.106	0.225
Serum nicotine, ng/mL	3.9 [1.7–8.2]	5.5 [4.2–7.4]	0 [0–0.7]		0 [0–0]		**0.025**		

Data are the median [interquartile range]. BSL, baseline; CC16, club cell protein-16; RBP, retinol binding protein; SP-D, surfactant protein-D; T60MIN, 60 min after exposure. *P* value in bold denotes the comparison between serum nicotine after vaping with nicotine compared with baseline.

### Effects of a Short-Term E-Cigarette Cessation on Baseline Serum and Urine Metabolome

**Fig. 8.**
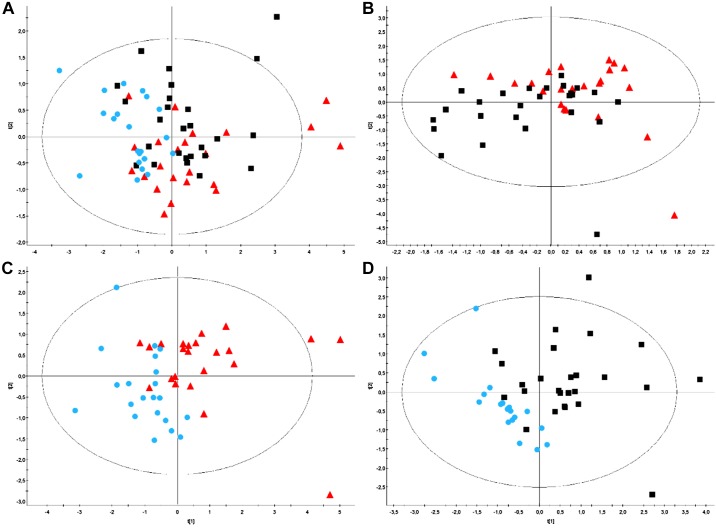
*A*–*D*: partial least squares discriminant analysis (PLS-DA) on baseline urine spectra. Patient clusters defined by classes: nicotine-session (red triangles), nicotine-free-session (black squares), and stop-session (blue dots). PLS-DA performed on baseline urine metabolome allowed us to split up the nicotine- and the stop-sessions (*C*) and the nicotine-free- and the stop-sessions (*D*) but not the nicotine- from the nicotine-free-session (*B*) or the other 3 sessions (*A*). *A*: model parameters: R^2^Xcum = 0.247; R^2^Ycum = 0.211; Q2cum = −0.00412; Hotelling T2 = 0.95, Two proposed principal components. *B*: model parameters: R^2^Xcum = 0.266; R^2^Ycum = 0.257; Q^2^cum = −0.21; Hotelling’s T2 = 0.95, Two proposed principal components. *C*: model parameters: R^2^Xcum = 0.306; R^2^Ycum = 0.464; Q^2^cum = 0.135; Hotelling’s T2 = 0.95, two proposed principal components. *D*: model parameters: R^2^Xcum = 0.253; R^2^Ycum = 0.511; Q^2^cum = 0.155; Hotelling’s T2 = 0.95, two proposed principal components.

**Fig. 9.**
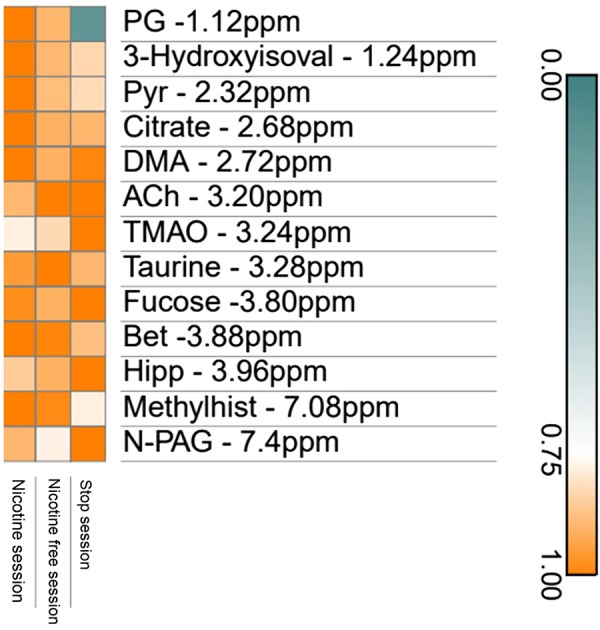
Heatmap plot using metabolites with variables of importance (VIP) values ≥ 1. 3-Hydroxyisoval, 3 – Hydroxyisovalerate; Ach, acethylcholine; Bet, betaine; DMA, dimethylamine; Hipp, hippurate; Methylhist, π-Methylhistidine; N-PAG, N- phenylacetylglycine; PG, propylene glycol; Pyr, pyruvate; TMAO, trimethylamine oxide.

PLS-DA performed on baseline serum metabolome did not allow us to split up any of the three experimental sessions. PLS-DA performed on baseline urine metabolome allowed to split up the nicotine and the stop sessions (Fig. 8*C*), the nicotine-free and the stop sessions (Fig. 8*D*), but not the nicotine from the nicotine-free-session (Fig. 8*B*) as well as all three sessions (Fig. 8*A*). Heatmap (Fig. 9) outlines the main metabolites with variables of importance values ≥ 1 of both sessions as well as those identified by the PLS-DA. Baseline urine propylene glycol was lower in the stop-session compared with the nicotine- and nicotine-free-sessions. Baseline urine 3-hydroxyisovalerate and urine pyruvate were higher in the nicotine session vs. the nicotine-free and the stop-sessions. Compared with the stop-session, baseline urine trimethylamine oxide and hippurate were lower in the nicotine-session. Finally, baseline N-Phenylacetyl-glycine was lower in the nicotine-session when compared with the stop-session. Supplemental Figure S2 shows representative 500-MHz-^1^HNMR spectra of urine samples from the three experimental sessions in the same subject.

## DISCUSSION

Our randomized crossover study presents these new findings: short-term e-cigarette cessation by regular users decreases baseline heart rate and lung inflammation and increases FEF-25%, suggesting that high-wattage vaping alters airway function. The urine metabolomic signature was also slightly modified by this short-term e-cigarette cessation. Acute nicotine- and nicotine-free-vaping decreased TcpO_2_, likely as a result of gas exchange disturbances. Finally, only acute nicotine-vaping increased SBP, DBP, and heart rate.

### Vaping Cessation Slightly Increases Serum CC16

Of interest is our observation that serum CC16 concentration increased after short-term e-cigarette cessation (stop-session). Current knowledge of serum CC16 kinetics after acute lung injury remains limited (30). Although the serum CC16 variations we observed were relatively small, large epidemiological studies have shown that serum CC16 variations of comparable magnitude are associated with more severe respiratory outcomes (28, 29). Low CC16 concentration in the serum has been associated with accelerated decline in forced expiratory volume in 1 s (FEV_1_) in chronic obstructive pulmonary disease (COPD) patients and development of moderate airflow limitation in the general population (28). Moreover, a low serum CC16 concentration can predict mortality risk, predominantly driven by lung cancer, in the general adult population (29). Recurrent exposure to noxious environmental factors, such as cigarette smoke, results in a decrease of serum CC16 (7, 69). Recently, the gene encoding for the CC16 protein has been found to be one of the top hypermethylated genes in airway epithelial cells in smokers compared with nonsmokers. This possibly supports an epigenetic mechanism for the shutdown of CC16 gene expression by chronic exposure to cigarette smoke (7, 69). Serum CC16 concentration increases after giving up tobacco (69), as also seen in the present study with vaping, suggesting that vaping triggers inflammation in the small airways (10, 12). This is also consistent with the lung inflammation we observed previously following acute vaping in naïve vapers (10, 12).

### Vaping and Lung-Function Tests

Whereas short-term vaping cessation seemed to improve the lung’s inflammation profile, it did not modify spirometry variables associated with pulmonary morbidity and mortality, such as FEV_1_ or lung-diffusion capacity (55). The diffusion capacity of the pulmonary membrane and the pulmonary capillary volume also remained unaffected by short-term vaping cessation (stop-session). FEF-25% was the only functional variable that slightly improved after 5 days of e-cigarette cessation compared with nicotine-free-vaping. The significance of the reduction in only FEF-25% is unclear but suggestive of changes in overall airway function. Although this change is probably clinically irrelevant, this observation is in line with our previous finding that acute vaping induced airway constriction in naïve vapers (10, 12). Although scant data exist on FEF, it appeared to be lower in tobacco smokers (26). It has been shown that spirometry and diffusing capacity improved 6 wk after cessation of tobacco smoking. However, there was no further improvement in these variables 1 yr later, suggesting irreversible lung lesions induced by tobacco smoke (17). Although our participants stopped tobacco smoking more than two years ago, their FEF-25% improved after short-term e-cigarette cessation. This FEF-25% improvement, together with a serum CC16 increase, suggests that chronic aerosol inhalation can have deleterious effects on the lung. Surprisingly, nicotine-vaping did not decrease FEF-25% compared with e-cigarette cessation in our study. Because of its properties as an airway irritant (38), nicotine could act as a safeguard to prevent excessive daily vehicle/aroma consumption during the 5 days before the experimental sessions (14). This safeguarding action could also modify the depth of inhalation and thus the amount and area of aerosol deposits in the lungs (36). This could also explain the absence of a superimposed effect on TcpO_2_ in cases of acute nicotine-vaping compared with acute nicotine-free-vaping.

### Acute Vaping Decreases Transcutaneous Oxygen Tension

Only a slight decrease in TcpO_2_ was observed in regular vapers in this study compared with the striking decrease previously shown in naïve e-cigarette-users after vaping (10, 12). This could result from adaptation of the lung to chronic exposure or relate to the smaller dose inhaled and the shorter puff time (3 s vs. 4 s) and vapor retention time (2 s vs. 4 s) in the present study (3). As in our previous investigations, the decrease in TcpO_2_ observed here was not accompanied by skin microcirculatory constriction, suggesting that vaping decreased arterial O_2_ partial pressure (10, 12). This is likely due to transient lung gas exchange disturbances induced by vaping (12, 48). Despite the lack of sensitivity of SpO_2_ to small changes in arterial O_2_ partial pressure in the anticipated ranges (33), it correlated with TcpO_2_ before and after acute exposure in this study, further indicating potential disturbances in lung gas exchanges caused by vaping (12, 33). Also, as previously shown, acute nicotine-vaping did not further decrease TcpO_2_ compared with nicotine-free-vaping (10, 12). Thus, the effects of vaping on TcpO_2_ appear largely attributable to propylene glycol and glycerol rather than nicotine. The small changes in TcPO_2_ we observed after acute vaping certainly do not have an acute clinical significance. However, these modifications could reflect, as previously postulated (12), airway collapses (ventilation/perfusion mismatches). We think that in case of repeated exposure (20 or 30 times per day) (35) over many years, this small phenomenon could be deleterious for the lungs. For instance, short-term effects of air pollution also induce very small variations in blood oxygenation without acute clinical significance (16, 40). However, long-term effects of air pollution are known to induce serious lung diseases, such as COPD (39). The large amount of hyperosmolar/hygroscopic propylene glycol/glycerol aerosol released during high-wattage vaping (19, 61) could induce inflammation and alter the properties of mucus/surfactant (31, 47, 50, 52, 58, 59), with subsequent acute transient perturbations in pulmonary gas exchange due to the underlying presence of areas with low ventilation-to-perfusion ratios comparable to those induced by nebulized hypertonic-saline and mannitol challenge tests (43). Finally, only acute nicotine-vaping induced a sustained TcpCO_2_ decrease, likely as a result of an increase in minute ventilation induced by nicotine, as previously observed in naïve e-cigarette-users (12).

### Nicotine-Vaping Modifies Hemodynamic Parameters

Only acute nicotine-vaping increased SBP, DBP, and heart rate compared with nicotine-free-vaping and sham-vaping. This is in accordance with known cardiovascular effects of nicotine resulting from activation of the sympathetic nervous system and subsequent chronotropic and inotropic effects (11). In our study, short-term e-cigarette cessation (stop-session) and nicotine-free-vaping also decreased baseline heart rate compared with nicotine-vaping. This is the first study to demonstrate that nicotine-cessation decreases heart rate in e-cigarette-users. Over 10% of users vape without nicotine when they give up tobacco (11, 57). In this small subpopulation of nicotine-free vapers, this may therefore decrease baseline heart rate and improve cardiovascular risk profile (22).

### Compliance to the Study Protocol

As nicotine and propylene glycol have a short serum half-life (2 and 4 h, respectively), their serum measurement was insufficient to certify that vaping had been stopped for 5 days (4, 20, 21). In contrast, urine cotinine has a long half-life (16–19 h) (32), and one recent study showed that propylene glycol was eliminated relatively slowly and persisted in the urine for up to 3 days after vaping (37). Altogether, it is reasonable to assume that measurements of serum nicotine and propylene glycol combined with urine cotinine and propylene glycol ascertain that the majority of our participants complied with the protocol. The modifications in urine metabolomics signature also argue in favor of sustained behavioral changes in our short-term vaping cessation versus pursuit groups similar to those seen after major changes, such as 4 days of bariatric surgery (24) or after 4 days (6) and 30 days (25) of smoking cessation (8, 56). The urine metabolomics variations we observed need to be confirmed by targeted metabolomics analyses and assessed in cell and animal models (9, 41, 67, 68). It should be noted that three participants in the nicotine-free-session and one other in the stop-session had abnormally high levels of serum nicotine and urine cotinine, which likely reflected a breach of the protocol but could also be explained by a slower nicotine metabolism in these four participants or secondhand tobacco exposure (not controlled for in this study) (4, 20).

### Limitations

This is the first study to test the effects of short-term e-cigarette cessation on regular heavy users. Although the vaping weaning period was short, most of the biological/clinical cardiorespiratory parameters we assessed shifted toward a healthier profile. There are some limitations, however. First, we did not monitor vaping conditions during the 5 days before the experimental sessions. Each e-cigarette device emits a specific profile of heavy metals, carbonyls, and even carbon monoxide (CO) (19, 61); all these are able to modify the absorbance properties of hemoglobin (33). The latter phenomenon could explain why baseline SpO_2_ was higher in the nicotine- and nicotine-free-sessions compared with the stop-session. It should be noted that although baseline CO did not correlate with baseline $\text{S}{\text{p}}_{{\text{O}}_{2}}$, it tended to be higher in the active vaping sessions compared with the stop-session, suggesting that in some cases high-wattage vaping can induce combustion rather than simple vaporization (19, 61). This could also modify $\text{S}{\text{p}}_{{\text{O}}_{2}}$absorbance (33). Because this study enrolled only male participants, our results should be replicated in female particpants, who may exhibit other vaping behaviors (51). Finally, as participants were former tobacco smokers, baseline $\text{S}{\text{p}}_{{\text{O}}_{2}}$, DLCO, and DLNO were abnormally low relative to age (34, 64). In future studies, these exclusive e-cigarette-users should also undergo serial lung-function tests and computed tomography to quantify the possible progression of lung-diffusion abnormality and detect lung emphysema and its progression under chronic vaping exposure. The present results should also be confirmed in regular vapers without a past history of tobacco smoking.

### Conclusions

In conclusion, our study reveals that short-term e-cigarette cessation in regular users decreases baseline heart rate and increases CC16 and FEF-25%, suggesting a slight improvement of airway status. Five days of vaping cessation also modified the urine metabolomic signature. Acute nicotine- and nicotine-free-vaping decreased TcpO2, likely as a result of transient lung gas exchange disturbances. Finally, only acute nicotine-vaping increases SBP, DBP, and heart rate.

## GRANTS

This study was supported by the “Fonds Erasme pour la Recherche Médicale,” Belgium (to M. Chaumont); the “Fonds Simone et Désiré Drieghe-Miller,” Belgium (to M. Chaumont); the “Fondation pour la Chirurgie Cardiaque,” Belgium (to M. Chaumont); the “Fondation Emile Saucez-René Van Poucke,” Belgium (to M. Chaumont); the “Prix Docteur & Mrs Rene Tagnon,” Belgium (to M. Chaumont); the “Fondation IRIS,” Belgium (to M. Chaumont); the “Prix de l’Association André Vésale,” Belgium (to M. Chaumont); a research grant of Astra Zeneca, Belgium (to P. van de Borne); the “Fonds Fruit de Deux Vies,” Belgium (to P. van de Borne); and the “Fond David and Alice Van Buuren,” Belgium (to P. van de Borne).

## DISCLOSURES

No conflicts of interest, financial or otherwise, are declared by the authors.

## AUTHOR CONTRIBUTIONS

M.C. and P.v.d.B. conceived and designed research; M.C., E.M.C., A.B., S.M., G.D., T.S., and V.F. performed experiments; M.C., V.T., J.-M.C., and A.V.M. analyzed data; M.C. and V.T. interpreted results of experiments; M.C. and V.T. prepared figures; M.C. and J.-M.C. drafted manuscript; M.C. and P.v.d.B. edited and revised manuscript; M.C., V.T., E.M.C., J.-M.C., A.B., S.M., G.D., A.V.M., N.D., T.S., V.F., and P.v.d.B. approved final version of manuscript.

## References

[1] Andersson L, Lundberg PA, Barregard L Methodological aspects on measurement of Clara cell protein in urine as a biomarker for airway toxicity, compared with serum levels. J Appl Toxicol 27: 60–66, 2007. doi:10.1002/jat.1184. 17186574

[2] Miller MR, Hankinson J, Brusasco V, Burgos F, Casaburi R, Coates A, Crapo R, Enright P, van der Grinten CP, Gustafsson P, Jensen R, Johnson DC, MacIntyre N, McKay R, Navajas D, Pedersen OF, Pellegrino R, Viegi G, Wanger J; ATS/ERS Task Force Standardisation of spirometry. Eur Respir J 26: 319–338, 2005. doi:10.1183/09031936.05.00034805. 16055882

[3] Beauval N, Verrièle M, Garat A, Fronval I, Dusautoir R, Anthérieu S, Garçon G, Lo-Guidice JM, Allorge D, Locoge N Influence of puffing conditions on the carbonyl composition of e-cigarette aerosols. Int J Hyg Environ Health 222: 136–146, 2019. doi:10.1016/j.ijheh.2018.08.015. 30220464

[4] Benowitz NL, Hukkanen J, Jacob P III Nicotine chemistry, metabolism, kinetics and biomarkers. In: Nicotine Psychopharmacology. Handbook of Experimental Pharmacology, edited by Henningfield JE, London ED, Pogun S Berlin: Springer, 2009, vol. 192, p. 29–60. doi:10.1007/978-3-540-69248-5_2. PMC2953858 19184645

[5] Bracci M, Ciarapica V, Copertaro A, Barbaresi M, Manzella N, Tomasetti M, Gaetani S, Monaco F, Amati M, Valentino M, Rapisarda V, Santarelli L Peripheral skin temperature and circadian biological clock in shift nurses after a day off. Int J Mol Sci 17: 623, 2016. doi:10.3390/ijms17050623. 27128899 PMC4881449

[6] Brown CR, Jacob P III, Wilson M, Benowitz NL Changes in rate and pattern of caffeine metabolism after cigarette abstinence. Clin Pharmacol Ther 43: 488–491, 1988. doi:10.1038/clpt.1988.63. 3365914

[7] Buro-Auriemma LJ, Salit J, Hackett NR, Walters MS, Strulovici-Barel Y, Staudt MR, Fuller J, Mahmoud M, Stevenson CS, Hilton H, Ho MW, Crystal RG Cigarette smoking induces small airway epithelial epigenetic changes with corresponding modulation of gene expression. Hum Mol Genet 22: 4726–4738, 2013. doi:10.1093/hmg/ddt326. 23842454 PMC3888123

[8] Bush T, Lovejoy JC, Deprey M, Carpenter KM The effect of tobacco cessation on weight gain, obesity, and diabetes risk. Obesity (Silver Spring) 24: 1834–1841, 2016. doi:10.1002/oby.21582. 27569117 PMC5004778

[9] Carrola J, Rocha CM, Barros AS, Gil AM, Goodfellow BJ, Carreira IM, Bernardo J, Gomes A, Sousa V, Carvalho L, Duarte IF Metabolic signatures of lung cancer in biofluids: NMR-based metabonomics of urine. J Proteome Res 10: 221–230, 2011. doi:10.1021/pr100899x. 21058631

[10] Chaumont M, Bernard A, Pochet S, Mélot C, El Khattabi C, Reye F, Boudjeltia KZ, Van Antwerpen P, Delporte C, van de Borne P High-wattage e-cigarettes induce tissue hypoxia and lower airway injury: a randomized clinical trial. Am J Respir Crit Care Med 198: 123–126, 2018. doi:10.1164/rccm.201711-2198LE. 29451806

[11] Chaumont M, de Becker B, Zaher W, Culié A, Deprez G, Mélot C, Reyé F, Van Antwerpen P, Delporte C, Debbas N, Boudjeltia KZ, van de Borne P Differential effects of e-cigarette on microvascular endothelial function, arterial stiffness and oxidative stress: a randomized crossover trial. Sci Rep 8: 10378, 2018. doi:10.1038/s41598-018-28723-0. 29991814 PMC6039507

[12] Chaumont M, van de Borne P, Bernard A, Van Muylem A, Deprez G, Ullmo J, Starczewska E, Briki R, de Hemptinne Q, Zaher W, Debbas N Fourth generation e-cigarette vaping induces transient lung inflammation and gas exchange disturbances: results from two randomized clinical trials. Am J Physiol Lung Cell Mol Physiol 316: L705–L719, 2019. doi:10.1152/ajplung.00492.2018. 30724099 PMC6589591

[13] Core Team R. R: A Language and Environment for Statistical Computing. Vienna, Austria: R Foundation for Statistical Computing, 2019 http://www.R-project.org.

[14] Dawkins L, Cox S, Goniewicz M, McRobbie H, Kimber C, Doig M, Kośmider L ‘Real-world’ compensatory behaviour with low nicotine concentration e-liquid: subjective effects and nicotine, acrolein and formaldehyde exposure. Addiction 113: 1874–1882, 2018. doi:10.1111/add.14271. 29882257 PMC6150437

[15] Dawson EA, Low DA, Meeuwis IH, Kerstens FG, Atkinson CL, Cable NT, Green DJ, Thijssen DH Reproducibility of cutaneous vascular conductance responses to slow local heating assessed using seven-laser array probes. Microcirculation 22: 276–284, 2015. doi:10.1111/micc.12196. 25703861

[16] DeMeo DL, Zanobetti A, Litonjua AA, Coull BA, Schwartz J, Gold DR Ambient air pollution and oxygen saturation. Am J Respir Crit Care Med 170: 383–387, 2004. doi:10.1164/rccm.200402-244OC. 15142869

[17] Dhariwal J, Tennant RC, Hansell DM, Westwick J, Walker C, Ward SP, Pride N, Barnes PJ, Kon OM, Hansel TT Smoking cessation in COPD causes a transient improvement in spirometry and decreases micronodules on high-resolution CT imaging. Chest 145: 1006–1015, 2014. doi:10.1378/chest.13-2220. 24522562 PMC4011651

[18] Dressel H, Filser L, Fischer R, de la Motte D, Steinhaeusser W, Huber RM, Nowak D, Jörres RA Lung diffusing capacity for nitric oxide and carbon monoxide: dependence on breath-hold time. Chest 133: 1149–1154, 2008. doi:10.1378/chest.07-2388. 18263682

[19] El-Hellani A, Al-Moussawi S, El-Hage R, Talih S, Salman R, Shihadeh A, Saliba NA Carbon monoxide and small hydrocarbon emissions from sub-ohm electronic cigarettes. Chem Res Toxicol 32: 312–317, 2019. doi:10.1021/acs.chemrestox.8b00324. 30656934

[20] Feyerabend C, Ings RM, Russel MA Nicotine pharmacokinetics and its application to intake from smoking. Br J Clin Pharmacol 19: 239–247, 1985. doi:10.1111/j.1365-2125.1985.tb02637.x. 3986082 PMC1463714

[21] Fowles JR, Banton MI, Pottenger LH A toxicological review of the propylene glycols. Crit Rev Toxicol 43: 363–390, 2013. doi:10.3109/10408444.2013.792328. 23656560

[22] Fox K, Borer JS, Camm AJ, Danchin N, Ferrari R, Lopez Sendon JL, Steg PG, Tardif JC, Tavazzi L, Tendera M; Heart Rate Working Group Resting heart rate in cardiovascular disease. J Am Coll Cardiol 50: 823–830, 2007. doi:10.1016/j.jacc.2007.04.079. 17719466

[23] Frank MS, Nahata MC, Hilty MD Glycerol: a review of its pharmacology, pharmacokinetics, adverse reactions, and clinical use. Pharmacotherapy 1: 147–160, 1981. doi:10.1002/j.1875-9114.1981.tb03562.x. 6927604

[24] Friedrich N, Budde K, Wolf T, Jungnickel A, Grotevendt A, Dressler M, Völzke H, Blüher M, Nauck M, Lohmann T, Wallaschofksi H Short-term changes of the urine metabolome after bariatric surgery. OMICS 16: 612–620, 2012. doi:10.1089/omi.2012.0066. 23095112

[25] Goettel M, Niessner R, Mueller D, Scherer M, Scherer G, Pluym N Metabolomic fingerprinting in various body fluids of a diet-controlled clinical smoking cessation study using a validated GC-TOF-MS metabolomics platform. J Proteome Res 16: 3491–3503, 2017. doi:10.1021/acs.jproteome.7b00128. 28849940

[26] Gold DR, Wang X, Wypij D, Speizer FE, Ware JH, Dockery DW Effects of cigarette smoking on lung function in adolescent boys and girls. N Engl J Med 335: 931–937, 1996. doi:10.1056/NEJM199609263351304. 8782500

[27] Guelen I, Westerhof BE, Van Der Sar GL, Van Montfrans GA, Kiemeneij F, Wesseling KH, Bos WJ Finometer, finger pressure measurements with the possibility to reconstruct brachial pressure. Blood Press Monit 8: 27–30, 2003. doi:10.1097/00126097-200302000-00006. 12604933

[28] Guerra S, Halonen M, Vasquez MM, Spangenberg A, Stern DA, Morgan WJ, Wright AL, Lavi I, Tarès L, Carsin AE, Dobaño C, Barreiro E, Zock JP, Martínez-Moratalla J, Urrutia I, Sunyer J, Keidel D, Imboden M, Probst-Hensch N, Hallberg J, Melén E, Wickman M, Bousquet J, Belgrave DC, Simpson A, Custovic A, Antó JM, Martinez FD Relation between circulating CC16 concentrations, lung function, and development of chronic obstructive pulmonary disease across the lifespan: a prospective study. Lancet Respir Med 3: 613–620, 2015. doi:10.1016/S2213-2600(15)00196-4. 26159408 PMC4640928

[29] Guerra S, Vasquez MM, Spangenberg A, Halonen M, Martinez FD Serum concentrations of club cell secretory protein (Clara) and cancer mortality in adults: a population-based, prospective cohort study. Lancet Respir Med 1: 779–785, 2013. doi:10.1016/S2213-2600(13)70220-0. 24461757 PMC3984132

[30] Hermans C, Bernard A Lung epithelium-specific proteins: characteristics and potential applications as markers. Am J Respir Crit Care Med 159: 646–678, 1999. doi:10.1164/ajrccm.159.2.9806064. 9927386

[31] Iskandar AR, Gonzalez-Suarez I, Majeed S, Marescotti D, Sewer A, Xiang Y, Leroy P, Guedj E, Mathis C, Schaller JP, Vanscheeuwijck P, Frentzel S, Martin F, Ivanov NV, Peitsch MC, Hoeng J A framework for in vitro systems toxicology assessment of e-liquids. Toxicol Mech Methods 26: 392–416, 2016. doi:10.3109/15376516.2016.1170251. 27117495 PMC5309872

[32] Jarvis MJ, Russell MA, Benowitz NL, Feyerabend C Elimination of cotinine from body fluids: implications for noninvasive measurement of tobacco smoke exposure. Am J Public Health 78: 696–698, 1988. doi:10.2105/AJPH.78.6.696. 3369603 PMC1350287

[33] Jubran A Pulse oximetry. Crit Care 19: 272, 2015. doi:10.1186/s13054-015-0984-8. 26179876 PMC4504215

[34] Kirby M, Owrangi A, Svenningsen S, Wheatley A, Coxson HO, Paterson NA, McCormack DG, Parraga G On the role of abnormal DL_CO_ in ex-smokers without airflow limitation: symptoms, exercise capacity and hyperpolarised helium-3 MRI. Thorax 68: 752–759, 2013. doi:10.1136/thoraxjnl-2012-203108. 23604381

[35] Kośmider L, Jackson A, Leigh N, O’Connor R, Goniewicz ML Circadian puffing behavior and topography among e-cigarette users. Tob Regul Sci 4: 41–49, 2018. doi:10.18001/TRS.4.5.4. 30778393 PMC6377077

[36] Labiris NR, Dolovich MB Pulmonary drug delivery. Part I: physiological factors affecting therapeutic effectiveness of aerosolized medications. Br J Clin Pharmacol 56: 588–599, 2003. doi:10.1046/j.1365-2125.2003.01892.x. 14616418 PMC1884307

[37] Landmesser A, Scherer M, Pluym N, Sarkar M, Edmiston J, Niessner R, Scherer G Biomarkers of exposure specific to e-vapor products based on stable-isotope labeled ingredients. Nicotine Tob Res 21: 314–322, 2019. doi:10.1093/ntr/nty204. 30265341

[38] Lee LY, Lin RL, Khosravi M, Xu F Reflex bronchoconstriction evoked by inhaled nicotine aerosol in guinea pigs: role of the nicotinic acetylcholine receptor. J Appl Physiol (1985) 125: 117–123, 2018. doi:10.1152/japplphysiol.01039.2017. 29369741 PMC6086971

[39] Liu C, Chen R, Sera F, Vicedo-Cabrera AM, Guo Y, Tong S, Coelho MS, Saldiva PH, Lavigne E, Matus P, Valdes Ortega N, Osorio Garcia S, Pascal M, Stafoggia M, Scortichini M, Hashizume M, Honda Y, Hurtado-Díaz M, Cruz J, Nunes B, Teixeira JP, Kim H, Tobias A, Íñiguez C, Forsberg B, Åström C, Ragettli MS, Guo YL, Chen BY, Bell ML, Wright CY, Scovronick N, Garland RM, Milojevic A, Kyselý J, , et al Ambient particulate air pollution and daily mortality in 652 cities. N Engl J Med 381: 705–715, 2019. doi:10.1056/NEJMoa1817364. 31433918 PMC7891185

[40] Luttmann-Gibson H, Sarnat SE, Suh HH, Coull BA, Schwartz J, Zanobetti A, Gold DR Short-term effects of air pollution on oxygen saturation in a cohort of senior adults in Steubenville, Ohio. J Occup Environ Med 56: 149–154, 2014. doi:10.1097/JOM.0000000000000089. 24451609 PMC3987810

[41] Madsen CT, Sylvestersen KB, Young C, Larsen SC, Poulsen JW, Andersen MA, Palmqvist EA, Hey-Mogensen M, Jensen PB, Treebak JT, Lisby M, Nielsen ML Biotin starvation causes mitochondrial protein hyperacetylation and partial rescue by the SIRT3-like deacetylase Hst4p. Nat Commun 6: 7726, 2015. doi:10.1038/ncomms8726. 26158509 PMC4510963

[42] Morpheus Morpheus (Online). https://software.broadinstitute.org/morpheus

[43] Muñoz PA, Gómez FP, Manrique HA, Roca J, Barberà JA, Young IH, Anderson SD, Rodríguez-Roisin R Pulmonary gas exchange response to exercise- and mannitol-induced bronchoconstriction in mild asthma. J Appl Physiol (1985) 105: 1477–1485, 2008. doi:10.1152/japplphysiol.00108.2008. 18756011

[44] National Academies of Sciences, Engineering, and Medicine Public Health Consequences of E-Cigarettes, edited by Stratton K, Kwan LY, Eaton DL Washington, DC: The National Academies Press, 2018. doi:10.17226/24952. 29894118

[45] Nishiyama T, Nakamura S, Yamashita K Effects of the electrode temperature of a new monitor, TCM4, on the measurement of transcutaneous oxygen and carbon dioxide tension. J Anesth 20: 331–334, 2006. doi:10.1007/s00540-006-0422-9. 17072703

[46] Orton DJ, Boyd JM, Affleck D, Duce D, Walsh W, Seiden-Long I One-step extraction and quantitation of toxic alcohols and ethylene glycol in plasma by capillary gas chromatography (GC) with flame ionization detection (FID). Clin Biochem 49: 132–138, 2016. doi:10.1016/j.clinbiochem.2015.09.007. 26385496

[47] Palazzolo DL, Nelson JM, Ely EA, Crow AP, Distin J, Kunigelis SC The effects of electronic cigarette (ECIG)-generated aerosol and conventional cigarette smoke on the mucociliary transport velocity (MTV) using the bullfrog (*R. catesbiana)* palate paradigm. Front Physiol 8: 1023, 2017. doi:10.3389/fphys.2017.01023. 29321743 PMC5732188

[48] Petersson J, Glenny RW Gas exchange and ventilation-perfusion relationships in the lung. Eur Respir J 44: 1023–1041, 2014. doi:10.1183/09031936.00037014. 25063240

[49] Phillips B, Esposito M, Verbeeck J, Boué S, Iskandar A, Vuillaume G, Leroy P, Krishnan S, Kogel U, Utan A, Schlage WK, Bera M, Veljkovic E, Hoeng J, Peitsch MC, Vanscheeuwijck P Toxicity of aerosols of nicotine and pyruvic acid (separate and combined) in Sprague-Dawley rats in a 28-day OECD 412 inhalation study and assessment of systems toxicology. Inhal Toxicol 27: 405–431, 2015. doi:10.3109/08958378.2015.1046000. 26295358

[50] Phillips B, Titz B, Kogel U, Sharma D, Leroy P, Xiang Y, Vuillaume G, Lebrun S, Sciuscio D, Ho J, Nury C, Guedj E, Elamin A, Esposito M, Krishnan S, Schlage WK, Veljkovic E, Ivanov NV, Martin F, Peitsch MC, Hoeng J, Vanscheeuwijck P Toxicity of the main electronic cigarette components, propylene glycol, glycerin, and nicotine, in Sprague-Dawley rats in a 90-day OECD inhalation study complemented by molecular endpoints. Food Chem Toxicol 109: 315–332, 2017. doi:10.1016/j.fct.2017.09.001. 28882640

[51] Piñeiro B, Correa JB, Simmons VN, Harrell PT, Menzie NS, Unrod M, Meltzer LR, Brandon TH Gender differences in use and expectancies of e-cigarettes: online survey results. Addict Behav 52: 91–97, 2016. doi:10.1016/j.addbeh.2015.09.006. 26406973 PMC4644488

[52] Przybyla RJ, Wright J, Parthiban R, Nazemidashtarjandi S, Kaya S, Farnoud AM Electronic cigarette vapor alters the lateral structure but not tensiometric properties of calf lung surfactant. Respir Res 18: 193, 2017. doi:10.1186/s12931-017-0676-9. 29149889 PMC5693547

[53] Restrepo RD, Hirst KR, Wittnebel L, Wettstein R AARC clinical practice guideline: transcutaneous monitoring of carbon dioxide and oxygen: 2012. Respir Care 57: 1955–1962, 2012. doi:10.4187/respcare.02011. 23107301

[54] Richard V, Conotte R, Mayne D, Colet JM Does the 1H-NMR plasma metabolome reflect the host-tumor interactions in human breast cancer? Oncotarget 8: 49915–49930, 2017. doi:10.18632/oncotarget.18307. 28611296 PMC5564817

[55] Schünemann HJ, Dorn J, Grant BJ, Winkelstein W Jr, Trevisan M Pulmonary function is a long-term predictor of mortality in the general population: 29-year follow-up of the Buffalo Health Study. Chest 118: 656–664, 2000. doi:10.1378/chest.118.3.656. 10988186

[56] Sealey WM, Teague AM, Stratton SL, Mock DM Smoking accelerates biotin catabolism in women. Am J Clin Nutr 80: 932–935, 2004. doi:10.1093/ajcn/80.4.932. 15447901 PMC1450014

[57] Smets J, Baeyens F, Chaumont M, Adriaens K, Van Gucht D When less is more: vaping low-nicotine vs. high-nicotine e-liquid is compensated by increased wattage and higher liquid consumption. Int J Environ Res Public Health 16: 723, 2019. doi:10.3390/ijerph16050723. 30823395 PMC6427796

[58] Sosnowski TR, Jabłczyńska K, Odziomek M, Schlage WK, Kuczaj AK Physicochemical studies of direct interactions between lung surfactant and components of electronic cigarettes liquid mixtures. Inhal Toxicol 30: 159–168, 2018. doi:10.1080/08958378.2018.1478916. 29932004

[59] Sosnowski TR, Odziomek M Particle size dynamics: toward a better understanding of electronic cigarette aerosol interactions with the respiratory system. Front Physiol 9: 853, 2018. doi:10.3389/fphys.2018.00853. 30038580 PMC6046408

[60] Tagliatti V, Colet JM Drug-induced impairment of mitochondrial fatty acid bet-oxidation: a metabolomic evaluation in rats. J Med Genomics 3: 005, 2016.

[61] Talih S, Salman R, Karaoghlanian N, El-Hellani A, Saliba N, Eissenberg T, Shihadeh A “Juice Monsters”: sub-ohm vaping and toxic volatile aldehyde emissions. Chem Res Toxicol 30: 1791–1793, 2017. doi:10.1021/acs.chemrestox.7b00212. 28937746

[62] Treml B, Kleinsasser A, Stadlbauer KH, Steiner I, Pajk W, Pilch M, Burtscher M, Knotzer H Cutaneous microvascular blood flow and reactivity in hypoxia. Front Physiol 9: 160, 2018. doi:10.3389/fphys.2018.00160. 29559919 PMC5845666

[63] Van Miert E, Sardella A, Nickmilder M, Bernard A Respiratory effects associated with wood fuel use: a cross-sectional biomarker study among adolescents. Pediatr Pulmonol 47: 358–366, 2012. doi:10.1002/ppul.21554. 21901861

[64] Vold ML, Aasebø U, Melbye H Low FEV_1_, smoking history, and obesity are factors associated with oxygen saturation decrease in an adult population cohort. Int J Chron Obstruct Pulmon Dis 9: 1225–1233, 2014. doi:10.2147/COPD.S69438. 25364242 PMC4211871

[65] Wattel FE, Mathieu DM, Neviere RR Transcutaneous oxygen pressure measurements: a useful technique to appreciate the oxygen delivery to tissues. J. Hyperbaric Med 6: 269–282, 1991.

[66] Williams B, Mancia G, Spiering W, Agabiti Rosei E, Azizi M, Burnier M, Clement DL, Coca A, de Simone G, Dominiczak A, Kahan T, Mahfoud F, Redon J, Ruilope L, Zanchetti A, Kerins M, Kjeldsen SE, Kreutz R, Laurent S, Lip GYH, McManus R, Narkiewicz K, Ruschitzka F, Schmieder RE, Shlyakhto E, Tsioufis C, Aboyans V, Desormais I, De Backer G, Heagerty AM, Agewall S, Bochud M, Borghi C, Boutouyrie P; ESC Scientific Document Group 2018 ESC/ESH Guidelines for the management of arterial hypertension. Eur Heart J 39: 3021–3104, 2018. doi:10.1093/eurheartj/ehy339. 30165516

[67] Yap IK, Brown IJ, Chan Q, Wijeyesekera A, Garcia-Perez I, Bictash M, Loo RL, Chadeau-Hyam M, Ebbels T, De Iorio M, Maibaum E, Zhao L, Kesteloot H, Daviglus ML, Stamler J, Nicholson JK, Elliott P, Holmes E Metabolome-wide association study identifies multiple biomarkers that discriminate north and south Chinese populations at differing risks of cardiovascular disease: INTERMAP study. J Proteome Res 9: 6647–6654, 2010. doi:10.1021/pr100798r. 20853909 PMC3117148

[68] Yu D, Shu XO, Rivera ES, Zhang X, Cai Q, Calcutt MW, Xiang YB, Li H, Gao YT, Wang TJ, Zheng W Urinary levels of trimethylamine-N-oxide and incident coronary heart disease: a prospective investigation among urban Chinese adults. J Am Heart Assoc 8: e010606, 2019. doi:10.1161/JAHA.118.010606. 30606084 PMC6405718

[69] Zhu L, Di PY, Wu R, Pinkerton KE, Chen Y Repression of CC16 by cigarette smoke (CS) exposure. PLoS One 10: e0116159, 2015. doi:10.1371/journal.pone.0116159. 25635997 PMC4312097

